# Spectra Analysis and Plants Research 2.0

**DOI:** 10.3390/plants13202941

**Published:** 2024-10-21

**Authors:** Ioan Grozescu, Maria Iorizzi, Adina-Elena Segneanu

**Affiliations:** 1 CAICON Department, University Politehnica Timisoara, 300006 Timisoara, Romania; ioangrozescu@gmail.com; 2 Department of Biosciences and Territory, University of Molise, Contrada Fonte Lappone, 86090 Isernia, Italy; iorizzi@unimol.it; 3 Institute for Advanced Environmental Research-West, University of Timisoara (ICAM-WUT), Oituz nr. 4, 300223 Timisoara, Romania

Medicinal plants have held a crucial position throughout human history, used in ethnomedicine, food preparation, preservation, cosmetics, decoration, disinfection, repelling pests, fabric dyeing, fertility techniques, and spiritual rituals [1,2,3,4,5].

The results of recent chemical analyses of widely recognized medicinal plants have unveiled the intricate compositions of biomolecules, such as phenolic acids, flavonoids, terpenes, tannins, alkaloids, lignans, coumarins, amino acids and peptides, iridoids, fatty acids, phytosterols, nucleosides, glycosides, carbohydrates, alcohols, polyketides, phenylpropanoids, carotenoids [1,2,3,4,5]. Natural products derived from plants are the largest resource used for drug discovery in modern medicine, and over 50% of currently available FDA-approved drugs have been derived from natural products. The search for new synthetic drugs is a long, risky, and expensive process that is frequently unsuccessful.

Research has established the therapeutic potential of these plants, driving rigorous exploration for drug development, focusing on the identification, isolation, synthesis, or de novo design of pseudo-natural products using various bioactive molecules with distinct chemical structures as generative models. In addition to the pharmaceutical industry, natural products have found applications as nutraceuticals, as well as in food supplements and cosmetics, and other area. The biological properties of plants and their composition in secondary metabolites are closely correlated, so in recent years, these correlations have stimulated the development of new, more efficient methodologies for the extraction, isolation, and characterization of phytoconstituents [1,2,3,4,5,6].

The bioactive components in medicinal or edible plants can be recovered using various extraction techniques. These involve the separation of several categories of biomolecules from a complex matrix, which is followed by individual molecule separation and then characterization. In traditional extraction methods (such as digestion, maceration, percolation, decoction, and Soxhlet extraction), the process is based on the use of organic solvents with different polarity. The most modern techniques are more efficient, employing environmentally friendly and less harmful solvents; these techniques include microwave-assisted extraction (MAE), ultrasound-assisted extraction (UAE), supercritical fluid extraction (SFE), subcritical water extraction, solid phase micro extraction (SPME), and enzyme-assisted extraction (EAE), which are widely referred to as green extraction technologies. The next step involves the separation and purification of the target molecule from the extracts using chromatographic techniques.

Gas chromatography (GC) and gas chromatography–mass spectrometry (GC-MS), are the effective methods used for the separation of volatile or apolar components, whereas medium-polarity or polar compounds can be easily identified and quantified using liquid chromatography–mass spectrometry (LC-MS), ultra-high-performance liquid chromatography–mass spectrometry (UHPLC-MS), as well as their respective tandem mass spectrometry methods (GC-MS/MS, LC-MS/MS, and UHPLC-MS/MS).

HPLC is a versatile analytical technique applied to separate, identify and quantify compounds even from complex matrices. HPLC allows the use of chromatography columns with different stationary phases and can be exploited for analytical and/or preparative purposes. The commonly used detectors include UV–Vis photodiode array (PDA), fluorescence detectors, refractive index detectors (RIDs), and light-scattering detectors. [1,4].

A crucial step in these processes is the characterization of phytochemicals via determining their chemical structure. A comprehensive approach involving UV–visible absorption spectroscopy (UV–Vis), infrared spectroscopy (IR), nuclear magnetic resonance (NMR) spectroscopy, and mass spectrometry (MS) is employed to elucidate the structure of biomolecules, which is also useful for defining the structure of synthetic compounds [1,2,3,4,5,6,7].

UV–Vis method is a simple and rapid qualitative analysis method that requires the presence of chromophores and provides valuable information about phytoconstituents with an aromatic nucleus, hydroxyl groups, and unsaturated bonds, such as flavonoids and anthocyanins [1,4]. Fourier-transform infrared spectroscopy (FTIR) is a fast, nondestructive, and cost-efficient method for identifying the functional groups present in phytochemicals. FTIR is an invaluable tool for elucidating the structure of phytochemicals when combined with UV–Vis and MS [1,3,4,7].

Mass spectrometry, a crucial and highly sensitive technique, allows for the elucidation of the molecular weight, formula, and fragmentation structure of biomolecules [1,4]. The ionization source is preferably electrospray ionization (ESI) or matrix-assisted laser desorption ionization (MALDI), and the analyzer is commonly a quadrupole (Q), a time-of-flight (TOF), or an orbitrap analyzer. Many current MS systems are tandem mass spectrometry systems, also known as MS/MS systems, which more accurately identify the individual ions analyzed.

Compared with other spectroscopic tools, NMR provides more detailed information on chemical structure through the detection of ^1^H and ^13^C nucleus in the unknown compound, offering advantages in terms of simplicity of sample preparation, high reproducibility, and acquisition of large amounts of data in a relatively short time. One-dimensional experiments (^1^H, ^13^C, ^31^P, ^15^N, and ^19^F) and two-dimensional NMR experiments (such as COSY, TOCSY, HSQC, and HMBC) have been developed to identify molecules with complex chemical structures. An emerging technology is metabolomics, which aims to simultaneously detect all primary and secondary metabolites in a biological system and provides qualitative and quantitative information on its components. Metabolomics is therefore a powerful tool for defining the phytochemical profile in an extract for quality control, geographical discrimination, quantification, etc. [6]. Currently, the two main analytical techniques used for these purposes are nuclear magnetic resonance (NMR) spectroscopy, in both 1D and 2D experiments, and mass spectrometry (MS), often coupled with separation techniques such as liquid or gas chromatography (LC or GC, respectively) [6].

Many researchers have contributed to this Special Issue entitled Spectra. Analysis and Plant Research 2.0, consisting of 13 original articles, providing *Plants* readers with an up-to-date overview of the new perspectives and recent advances in the spectral techniques used for the investigation of natural products.

The papers submitted provide the results of studies conducted by research teams from Romania, Italy, Spain, Portugal, Czech Republic, Colombia, France, Germany, Slovakia, Chile, and Bulgaria, and cover different application areas ranging from food, biomedical, nutraceutical, biotechnological, and dietetics to cosmetics.

Cavallaro et al. [8] report an analysis of the essential oil from the roots, stems, leaves, and flowers of *Orchis purpurea* (genus *Orchis*), a herbaceous plant that is unpleasant to herbivorous animals. A total of 70 volatile components were identified via GC-FID analysis, which demonstrated a prevalence of coumarin. Nonvolatile fractions, obtained through Soxhlet extraction using three different solvents, were analyzed via UHPLC-MS, revealing the presence of hydroxycinnamic acid derivatives, polyphenols, and glycosidic compounds, which are probably responsible for their color and fragrance.

The composition of the volatile organic compounds of different rose essential oil samples (*Rosa damascene*, *Rosa alba*, *Rosa centifolia*, *Rosa gallica* (genus Rosa), and *Prasophyllum roseum* (genus Prasophyllum)) was analyzed by Koljančić et al. [9] using enantioselective two-dimensional gas chromatography coupled with high-resolution time-of-flight mass spectrometry (GC × GC–HRTOF-MS). The potential of separating the volatile compounds was evaluated with three different orthogonal combinations of stationary phases to classify different types of rose essential oils. The researchers also investigated the separation efficiencies of three stationary phases in the first dimension: Chirasil-Dex, MEGA-DEX DET—β, and Rt-βDEXsp. Chirasil-Dex. The results demonstrate the capability of tile-based Fisher ratio (F-ratio) chemometric analysis to reliably and rapidly identify and discriminate volatile components.

HPLC-DAD analysis combined with mass spectrometry was applied by Fernández-Galleguillos et al. [10] for the identification and quantification of the phenolic compounds from *Clinopodium gilliesii* (genus Clinopodium), *Mutisia acuminata* (genus Mutisia), and *Tagetes multiflora* (genus Tagetes). In total, 30 phenolic compounds were identified and quantified in all species using HPLC-DAD, whereas the other phenolic components were tentatively identified in the extracts via high-resolution ESI-MS-MS. The extracts were evaluated for their potential inhibitory effects against cholinesterases, which are the key enzymes linked to Alzheimer’s disease. The *T. multiflora* extract showed the highest anti-acetylcholinesterase (AChE) and anti-butyrylcholinesterase (BChE) activity. A molecular docking analysis was also performed to further understand the interactions between the cholinesterase enzymes and the main phenols identified in *T. multiflora*.

The phenolic metabolite profiles of three species of the Senecio genus (*Senecto hercynicus*, *Senecto ovatus*, and *Senecto rupestris*) and two species of Jacobaea (*J. pancicii* and *J. maritima*) were studied by Voynikov et al. [11]. A combination of morphometric and UHPLC-HRMS analyses was conducted. Using UHPLC-Orbitrap-MS, 46 phenolic metabolites were identified. The results of hierarchical and PCA clustering, which were applied to the phytochemical data, supported the similarity of *S. hercynicus* and *S. ovatus*, which was observed in the morphometric analysis.

Pedro et al. [12] examined the phenolic composition, as well as antioxidant and antimicrobial properties, of the extracts obtained from seven Acacia pod extracts (*Acacia melanoxylon*, *Aacacia longifolia*, *Acacia cyclops*, *Acacia retinodes*, *Acacia pycnantha*, *Acacia mearnsii*, and *Acacia dealbata*) to assess the potential industrial applications of the Acacia genus. Using LC-ESI-HRMS/MS, compounds belonging to different organic classes were identified and then quantitated via HPLC-DAD. The antioxidant properties of the samples were evaluated using the DPPH free radical scavenging assay and the b-carotene bleaching test, and antibacterial activity was assessed against *E. coli*, *K. pneumoniae*, *S. typhimurium*, and *B. cereus*.

Segneanu et al. [13,14] report a novel plant-based nanotechnology approach with the potential to become a high-performance therapeutic platform.

Their first paper [13] reports the low-molecular-mass-metabolite profiling of *Cheledonium majus* (genus Chelidonium), growing wild in Romania (via GC-MS and EIS-QTOF-MS), followed by the development and in vitro evaluation of the antioxidant and release of a novel carrier system prepared using this medicinal plant and AuNPs. Various analytical methods, including FTIR, Raman, XRD, DLS, and SEM-EDX, were employed to confirm the preparation of the carrier system. The results of the in vitro antioxidant assessment of the novel carrier system correlated to those of the medicinal plant.

In their second study, Segneanu et al. [14] investigated the low-molecular-weight metabolite profile of wild-grown Romanian *Helleborus purpurascens* Waldst. & Kit (genus Helleborus) (via GC-MS and electrospray ionization-quadrupole time-of-flight mass spectroscopy) and two new chitosan nanocarriers developed using this plant. The authors revealed the morpho-structural characteristics and thermal behavior of the newly prepared nanocarriers and their antioxidant potential [14].

Rodríguez et al. [15] analyzed the relationship between the dry matter (DM) content in whole Hass avocado using near-infrared spectroscopy (NIRS) scanning of different fruit components (peduncle, equator, and base) and at different ripeness levels. The results demonstrate the potential of using NIRS for the rapid selection of avocados according to their commercial quality.

Dima et al. [16] found that plant biostimulants enhance protection against abiotic stress and improve leaf dietary fiber composition. A combination of spectroscopic techniques (FTIR, XRD) and thermogravimetric analysis (TGs) was used to highlight the structural changes in the leaves of white cabbage (*Brassica oleracea var. capitata f. alba*, genus Brassica) treated with plant biostimulants (selenium–baker’s yeast vinasse formulation, Se-VF) included in a foliar fertilizer formula.

A metabolomic approach using ^1^H NMR fingerprinting and chemometrics was adopted by Samukha et al. [17] and Lanzotti et al. [18].

Samukha et al. [17] explored the hydroalcoholic and organic extracts from four different commercial *Phaseolus vulgaris* L. (*genus Phaseolus* L.) dry seed varieties using nontargeted and targeted chemometric analyses via ^1^H NMR-based metabolomics in combination with multivariate data analysis. More than 32 metabolites from various classes were identified, such as carbohydrates, amino acids, organic acids, nucleosides, alkaloids, and fatty acids. In addition, the total phenolic, total flavonoid, and condensed tannin contents were determined in ethanolic extracts, which was followed by the determination of the in vitro antioxidant potential (DPPH, ABTS, and FRAP assay) and their antifungal activity against the sclerotia growth of *S. rolfsii* [17].

Lanzotti et.al. [18] investigated the influence of an organic foliar treatment on the metabolic profiles of lettuce (*Lactuca sativa* L., *genus Lactuca* L.) in a field characterized by high infestation levels of the fungal pathogen *Fusarium oxysporum lactucae*. The composition of the secondary metabolites of the lettuce extracts was determined after each foliar treatment using ^1^H NMR-based metabolomics and chemometrics. The results of untargeted NMR metabolomics showed that the foliar organic application substantially altered the composition of several secondary metabolites; these results highlight the potential relevance of metabolomics approaches in the field of crop biostimulation and in the biocontrol of plant pathogens.

The proteomic profiles of whole, hulled, and decorticated seeds of two cultivars of industrial hemp (*Cannabis sativa* L., *genus Cannabis* L.) were obtained by Jan Bárta et al. [19]. Hemp seeds are often dehulled for consumption and food applications, removing the hard shells and thus increasing their nutritional value; however, the hulls become waste. The authors evaluated the proteome of hemp (*C. sativa* L.) for whole seeds, dehulled seeds, and hulls. Proteomic analysis was performed on two cultivars, Santhica 27 and Uso-31, using the LC-MS/MS technique, which enabled the identification of 2833 protein groups (PGs) and their relative abundances. Seed storage proteins were found to be the most abundant protein class; in particular, 11S globulins had a higher relative abundance in the dehulled seed proteome. The second most abundant class of proteins was oleosins. The hulls may therefore be an essential source of proteins, especially for medical and biotechnological applications.

Casal-Porras et al. [20] analyzed the correlation between the phenolic profile (rosmarinic acid and flavonoids) determined via UPLC-MS analysis, of *Zostera noltei* (*genus Zostera* L.) leaves (fresh, dried, and frozen leaves) (seagrasses) and the level of exposure to sunlight using an in situ experimental approach. Seagrasses are plants adapted to the marine environment that live in shallow coastal waters, where they may be exposed to direct sunlight during low tides. These plants have photoprotective mechanisms, which include the use of phenolic secondary metabolites. The rosmarinic acid content was considerably higher in plants that emerged during low tides than in plants permanently submerged. A positive correlation was found for rosmarinic acid with direct sunlight and UV exposure of the leaves, suggesting that this compound contributes to the photoprotection of *Z. noltei*. In addition, the variation in the phenolic profile was affected by the sample preparation method [10].

In summary, this Special Issue presents recent research findings on the correlation between the metabolic profiles of different plants and variables such as biotic and abiotic factors, growth stage, foliar organic fertilization treatments, and the extensive application potential of these natural compounds. Untargeted NMR metabolomics and proteomic approaches using the LC-MS/MS technique are promising tools for the analysis of metabolites in complex extracts. Future research should focus on assessing bioavailability and integrating nanotechnological tools to enhance their thermal and chemical stability and biological properties.
